# Colchicine Prevents Postoperative Atrial Fibrillation in Cardiac and Thoracic Surgery Patients: Contemporary Evidence From a Meta‐Analysis of Randomized Controlled Trials

**DOI:** 10.1155/crp/1851955

**Published:** 2026-06-17

**Authors:** Muhammad Hassan Saeed, Syeda Zaira Haider, Kishan Chand Lohana, Fnu Kashish, Muhammad Ahmed Zaheer, Tayyaba Naseem Abbasi, Safa Mazhar, Jarin Rahman

**Affiliations:** ^1^ Department of Medicine, Jinnah Postgraduate Medical Centre, Karachi, Pakistan, jpmc.edu.pk; ^2^ Department of Forensic Medicine, Al-Tibri Medical College, Isra University, Karachi, Pakistan, isra.edu.pk; ^3^ Department of Medicine, Jinnah Sindh Medical University, Karachi, Pakistan, jsmu.edu.pk; ^4^ Department of Surgery, Rajshahi Medical College Hospital, Rajshahi, Bangladesh

**Keywords:** cardiac surgery, colchicine, meta-analysis, postoperative atrial fibrillation, systematic review, thoracic surgery

## Abstract

**Background:**

Postoperative atrial fibrillation (POAF) is the most common arrhythmia following cardiac and thoracic surgery. Stems from atrial oxidative stress and pericardial inflammation, POAF is associated with adverse clinical outcomes such as heart failure, stroke, and increased mortality. As an anti‐inflammatory agent, colchicine has shown effective POAF risk reduction.

**Methods:**

A systematic review and meta‐analysis were conducted including randomized controlled trials (RCTs) evaluating colchicine for POAF prevention till september 2025. Data were pooled using random‐effects models with restricted maximum likelihood (REML). Subgroup analyses examined treatment duration (< 2 weeks vs. > 2 weeks) and surgery type. Heterogeneity was assessed with I^2,^ and stability of outcomes was assessed using leave‐one‐out (LOO) analyses.

**Results:**

Twelve RCTs encompassing 5637 participants were included. Colchicine significantly reduced POAF incidence compared with control (RR = 0.73; 95% CI = 0.64–0.84; *p* < 0.0001; I^2^ = 0%). Both short‐course (< 2 weeks) and long‐course (> 2 weeks) regimens were effective without any significant subgroup difference (*p* = 0.57). Colchicine increased gastrointestinal adverse events (OR = 2.25; 95% CI = 1.86–2.73) and diarrhea (RR = 3.16; 95% CI = 2.25–4.42) but showed no excess bleeding (RR = 0.89; 95% CI = 0.61–1.28), sepsis (RR = 1.32; 95% CI = 0.48–3.59), or in‐hospital mortality (RR = 0.90; 95% CI = 0.43–1.86). Funnel plots and Egger’s tests revealed no publication bias.

**Conclusions:**

Colchicine achieved significant POAF risk reduction without serious adverse events. Both short‐ and long‐course regimens were effective, with the short‐course approach offering optimal tolerability. These findings support colchicine as a safe, inexpensive adjunct for POAF prevention.

## 1. Introduction

Postoperative atrial fibrillation (POAF) is the most common arrhythmogenic outcome following cardiac surgery, with incidence rates ranging from 20% to 60%, according to the complexity and type of the procedure performed [1, 2]. This arrhythmia usually occurs within the first week after surgery and is most prevalent between postoperative Days 2–4 [3]. POAF is anything but benign; although it may often be self‐limited and transient, it is associated with a substantially greater risk for adverse clinical outcomes (i.e., heart failure, stroke, prolonged mechanical ventilation, extended hospital, and ICU length of stay) and increased healthcare costs, as well as both short and long‐term mortality [4]. POAF avoidance is an important objective in perioperative cardiovascular medicine, as these issues have a large burden on both the healthcare systems and patients.

Due to the complex interplay between structural, electrical, and metabolic changes in the atria, the pathophysiology of POAF is not yet completely elucidated. Autonomic instability, surgical trauma, atrial ischemia, oxidative stress, and intense systemic inflammatory response elicited by tissue injury and cardiopulmonary bypass are recognized as key pathophysiologic mechanisms [5, 6]. Atrial fibrillation occurs more in patients who are in a proinflammatory state because it promotes atrial remodeling, conduction abnormalities, and enhanced automaticity [7, 8]. Thus, anti‐inflammatory methods have been proposed as a method of retarding the onset of postoperative ischemic attack.

The widely used anti‐inflammatory medication colchicine, established to cure gout and pericarditis for decades, has been a potential POAF preventive agent. Its own pharmacological properties are NLRP3 inflammasome blocking, neutrophil chemotaxis suppression, and microtubule polymerization inhibition, all of which are significant mechanisms in the propagation of oxidative stress and inflammation after cardiac surgery [9–11]. Colchicine has been shown to reduce pericardial and atrial inflammation, inflammatory reactions after surgery, and finally, the incidence of atrial fibrillation by these mechanisms.

Preventive treatment with colchicine during cardiac surgery has been evaluated in various observational studies and randomized trials with mixed success [12]. While some randomized controlled trials (RCTs) have shown a significant decrease in the incidence of POAF and inflammatory biomarkers, others failed to produce these benefits but instead an increased incidence of gastrointestinal (GI) side effects, that is, nausea and diarrhea, which preclude its tolerability and usage on a daily basis [12, 13]. Existing synthesized evidence includes the recent meta‐analysis by Rivera et al. [14], which demonstrated a reduction in POAF incidence without significant impact on mortality, infection, or hospital stay. However, the emergence of additional RCTs and the availability of more detailed safety data, particularly regarding GI, infectious, and bleeding complications, warrant an updated evaluation.

This update meta‐analysis incorporates the latest randomized evidence to provide refined, evidence‐based estimates of colchicine’s efficacy and safety in the prevention of POAF among adult cardiac surgical patients. This comprehensive reassessment aims to clarify remaining uncertainties regarding the prophylactic role of colchicine, guide clinical decision‐making, and identify directions for future research.

## 2. Methodology

### 2.1. Study Design

This systematic review and meta‐analysis of RCTs was conducted as an update to Zhao et al.’s synthesis [14], assessed the efficacy and safety of colchicine in cardiac surgery patients. This review followed the Preferred Reporting Items for Systematic Reviews and Meta‐Analysis (PRISMA) 2020 guidelines [15] and is registered with the International Prospective Register of Systematic Reviews (PROSPERO) under the registration number: CRD420251152636 [16].

### 2.2. Eligibility Criteria

#### 2.2.1. Inclusion Criteria

Only RCTs involving adult patients (≥ 18 years) undergoing cardiac surgery, such as coronary artery bypass grafting (CABG), valve replacement or repair, or combined procedures, were included. Eligible studies were required to evaluate the use of colchicine administered perioperatively (before, during, or after surgery), at any dose, duration, or route of administration, compared with placebo or standard care. Only peer‐reviewed, full‐text RCTs published in English up to September 2025 were included.

#### 2.2.2. Exclusion Criteria

Studies were excluded if they were nonrandomized, observational in design, case reports, case series, reviews, editorials, or conference abstracts lacking full data. Animal or in vitro studies, trials not involving cardiac surgery patients, or studies where colchicine was used solely for indications unrelated to POAF prevention (such as gout or pericarditis) without reporting POAF outcomes were also excluded.

### 2.3. Outcomes

The primary outcome of interest was the incidence of POAF, while secondary outcomes included length of hospital stay, mortality rates, sepsis or infection, and adverse effects (particularly GI events, diarrhea, and bleeding).

### 2.4. Search Strategy

A thorough literature search was conducted across the following electronic databases: PubMed, Google Scholar, Scopus, Cochrane Library, and Embase. Additional clinical trials were searched on ClinicalTrials.gov for unpublished or ongoing trials. The bibliographies and reference lists of included studies, along with relevant review articles, were manually searched to identify additional eligible trials. The search strategy incorporated relevant Medical Subject Headings (MeSH) terms and keywords, including: “Postoperative Atrial Fibrillation” OR “POAF” OR “Atrial Fibrillation after Cardiac Surgery” OR “Post‐cardiac surgery arrhythmia” AND “Colchicine” OR “Anti‐inflammatory therapy” OR “NLRP3 inflammasome inhibitor” AND “Cardiac surgery” OR “Coronary artery bypass graft” OR “CABG” OR “Valve surgery” OR “Cardiopulmonary bypass.”

A complete search string for each database is provided in the supporting data.

### 2.5. Study Selection

Two reviewers performed the literature search, screening, and study selection independently. Titles and abstracts were screened for eligible studies; after that, full‐text review was performed, and articles were evaluated against eligibility criteria. Any conflicts or disagreements during screening were resolved through mutual discussion or by consulting a third reviewer. The PRISMA 2020 flow diagram was adopted to document the selection process [15].

### 2.6. Data Extraction

Two reviewers independently extracted data from each included RCT using a predefined extraction form developed in accordance with the PRISMA 2020 guidelines [15]. Extracted variables included study characteristics (first author, year of publication, study location, population, follow‐up duration, design, POAF monitoring, sample size, and intervention details), baseline characteristics (sex, age, BMI, diabetes, hypertension, smoking, concurrent medications, stroke, and previous AMI), and all predefined outcomes. When outcome data were missing or unclear, supporting information was requested directly from authors. Any conflicts or disagreements during screening were resolved through mutual discussion or by consulting a third reviewer.

### 2.7. Risk of Bias Assessment

Two reviewers independently assessed the quality of the included studies using the Revised Cochrane Risk‐of‐Bias Tool for Randomized Trials (RoB 2) [17]. Studies were assessed across the following five domains: bias arising from the randomization process, bias due to deviations from intended interventions, bias due to missing outcome data, bias in measurement of the outcome, and bias in selection of the reported results. Each domain was judged as “low risk,” “some concerns,” or “high risk” of bias, and an overall risk of bias judgment was assigned to each study. Any disagreements in rating were resolved through mutual discussion or by consulting a third reviewer. Risk of bias plots were generated using the ROBVIS online tool (Risk‐of‐Bias VISualization) [18].

### 2.8. Statistical Data Analysis Plan

Effect sizes for dichotomous outcomes were represented as risk ratios (RR) or odds ratios (OR) accompanied by corresponding 95% confidence intervals, utilizing a random effects model to accommodate potential heterogeneity. Subgroup analysis by treatment duration and type of surgery was also carried out. All analyses were performed using Review Manager (RevMan) Version 5.4, and R programming, and forest plots were generated.

### 2.9. Assessment of Heterogeneity

Statistical heterogeneity was assessed using the Chi‐square (*χ* ^2^) test (with *p* < 0.10 indicating significant heterogeneity) I^2^ statistic (an I^2^ value > 50% was considered substantial heterogeneity). Sensitivity analysis and leave‐one‐out (LOO) analysis were also performed to identify studies contributing significantly to overall heterogeneity. Egger’s regression test and Funnel plot were also employed to evaluate the publication bias. The Grading of Recommendations Assessment, Development and Evaluation (GRADE) approach was used to assess the certainty of evidence across studies for each outcome [19]. Where substantial heterogeneity was observed, predefined sensitivity and LOO analyses were undertaken; if heterogeneity was explained or resolved with a consistent direction of effect, inconsistency was not considered serious for GRADE assessment.

## 3. Results

### 3.1. Study Selection

**FIGURE 1:**
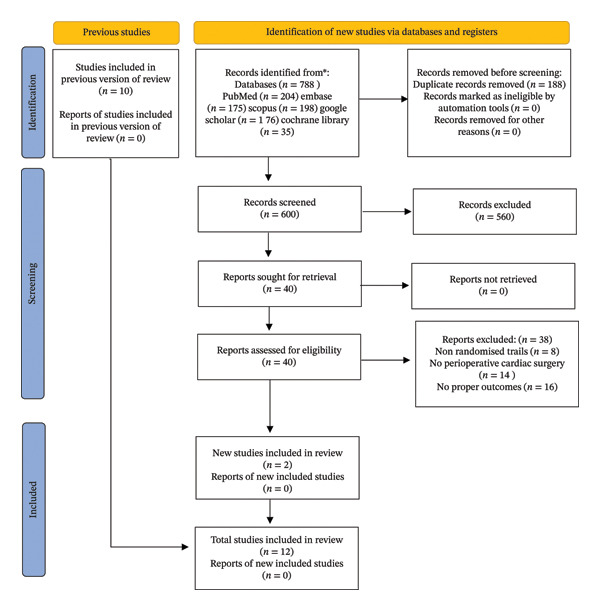
Search strategy and study selection process illustrated using the PRISMA 2020 flow diagram for an updated systematic review. After systematic screening and eligibility assessment, two additional randomized placebo‐controlled trials were included in the updated synthesis.

A total of 788 studies were identified through the initial literature search. After the removal of 188 duplicates, 600 articles were left for screening. Upon title and abstract review, 560 studies did not match our inclusion criteria and were excluded. The remaining 40 studies underwent full‐text evaluation. Ultimately, two RCTs met the eligibility criteria and were included in this updated meta‐analysis [20, 21]. The study selection process is summarized in the PRISMA 2020 flow diagram. (Figure 1).

### 3.2. Study and Baseline Characteristics

A total of 12 RCTs [20–31] comprising 5637 patients were included in this updated meta‐analysis. Among these, 41.2% were female. There were 2819 patients in the colchicine arm and 2818 in the placebo arm. The mean age across studies was 64.2 years, ranging from 62.3 years in Diakova et al. to 80.8 years in Ryffel et al. More than half of the participants (57%) had hypertension, while 23% had diabetes and 24% were active smokers. Among these, 3310 patients underwent thoracic procedures, predominantly lung resections (99%), with smaller subsets undergoing pneumonectomy, mediastinal mass resection, or decortication. The remaining patients underwent cardiac surgery, most commonly CABG (56%), followed by aortic (9%), valvular (7%), and combined cardiac procedures (20%). Across the included trials, colchicine was administered orally in all studies, most commonly at a dose of 0.5–1.0 mg twice daily (BID), initiated either 24 h before surgery or within the first postoperative day. The duration of treatment varied considerably, ranging from 7 to 30 days. A few studies implemented shorter courses during the in‐hospital stay only, while others continued colchicine therapy up to 4 weeks postdischarge. Follow‐up periods across trials ranged from 7 days to 1 month, depending on the index procedure and study design. Continuous ECG monitoring was used in most cardiac surgery trials, whereas daily or intermittent ECG recordings were applied in thoracic procedures. Importantly, clinically relevant differences existed across included populations, including CABG, valve surgery, thoracic surgery, and transcatheter aortic valve replacement (TAVR) cohorts, each with distinct arrhythmic substrates, inflammatory burdens, perioperative management strategies, and monitoring intensities.

The newly included RCT by Diakova et al. (2025) enrolled 140 patients undergoing isolated CABG under cardiopulmonary bypass, evaluating perioperative low‐dose colchicine combined with pericardial fenestration. The Ryffel et al. (2025) trial contributed 120 elderly patients (mean age = 80 years) undergoing TAVR. There were high baseline rates of hypertension (≥ 80%), diabetes (30%), and beta‐blocker use (38%).

Study and Baseline characteristics of the included trials are summarized in Tables 1 and 2.

**TABLE 1:** Study characteristics of the randomized controlled trials comparing colchicine versus placebo in patients with cardiac/thoracic surgery.

Study (first author, year)	Participants (*n*)			Population	Type of surgery	Location	Follow‐up duration	Intervention (dosage, duration)	Control
	Total	Colchicine	Placebo						
Imazio et al., 2011	360	180	180	Patients undergoing elective cardiac surgery	CABG, valvular, aortic, combined	Italy	1 month	1.0 mg BID starting on postoperative Day 3 and then 0.5 mg BID for 1 month	Placebo
Imazio et al., 2014	360	180	180	Patients undergoing elective cardiac surgery	CABG, valvular, aortic, combined	Italy	3 months	0.5 mg BID (0.5 mg OD if,70 kg) starting 2‐3 days before surgery for 1 month	Placebo
Sarzaeem et al., 2014	216	108	108	Patients undergoing elective cardiac surgery	CABG	Iran	In‐hospital stay	1 mg the night before surgery and on the morning of surgery, and then 0.5 mg BID for 5 days after surgery	Placebo
Tabbalat et al., 2016	360	179	181	Patients undergoing elective cardiac surgery	CABG, combined procedures	Jordan	In‐hospital stay	2 mg a day before surgery and 1 mg 4 h before or immediately after surgery, and then 0.5 mg BID until hospital discharge	Placebo
Zarpelon et al., 2016	140	71	69	Patients admitted for myocardial revascularization surgery	CABG	Brazil	In‐hospital stay	1 mg BID starting a day before surgery, followed by 0.5 mg BID until discharge	Placebo
Bessissow et al., 2017	100	49	51	Patients undergoing lung resection surgery	Patients undergoing tumor resection in the lung	Canada	1 month	0.6 mg of colchicine or placebo PO within 4 h before surgery, then another 0.6 mg as the second dose, and then 0.6 mg BID for 9 days	Placebo
Tabbalat et al., 2020	152	81	71	Patients undergoing elective cardiac surgery	CABG, valvular surgeries, combined surgery	Jordan	In‐hospital stay	1 mg 12–24 h before surgery, followed by 0.5 mg OD until discharge	Placebo
Masheyekhi et al., 2020	240	120	120	Patients undergoing open heart surgery	CABG, valvular disease, aortic disease	Iran	6 months	1 mg BID in D1, then 1 mg OD for 1 month (0.5 mg in patients, 70 kg)	Placebo
Shvartz et al., 2022	240	113	127	Patients undergoing open heart surgery	CABG or AVR	Russia	1 week	1 mg OD starting 1 day before surgery until 5 days	Placebo
Conen et al., 2023	3209	1608	1601	Patients undergoing major noncardiac thoracic surgery	Thoracic surgery	Canada	2 weeks	0.5. mg BID for 10 days	Placebo
Diakova et al., 2025 (CAFÉ)	140	70	70	Adults undergoing elective on‐pump cardiac surgery (CABG ± valve)	CABG ± valvular procedures	Russia	10 days	Colchicine 0.5 mg BID (started 24 h before surgery and continued for 10 days); combined with pericardial fenestration in all patients	Placebo
Ryffel et al., 2025 (Co‐STAR)	120	60	60	Patients with severe aortic stenosis undergoing transcatheter aortic valve replacement (TAVR)	TAVR (percutaneous cardiac procedure)	Switzerland	1 month	Colchicine 0.5 mg BID initiated 24 h before TAVR and continued for 13 days	Placebo

Abbreviations: AVR, aortic valve replacement; BID, Bis in die (twice daily); CABG, coronary artery bypass grafting; OD, Omni die (Once daily); PO, Per os (per oral).

**TABLE 2:** Baselines of the randomized controlled trials comparing colchicine versus placebo in patients with cardiac/thoracic surgery.

Study (first author, year)	Participants (*n*)			Mean age (years)		Female, *n* (%)		BMI (kg/m^2^)		Diabetes, *n* (%)		Hypertension, *n* (%)		Smoking, *n* (%)		Previous AMI, *n* (%)		Stroke, *n* (%)		Medications, *n* (%)	
	Total	Colchicine	Placebo	Colchicine	Placebo	Colchicine	Placebo	Colchicine	Placebo	Colchicine	Placebo	Colchicine	Placebo	Colchicine	Placebo	Colchicine	Placebo	Colchicine	Placebo	Colchicine	Placebo
Imazio et al., 2011	360	180	180	64.8 (13.7)	66.6 (11.0)	51 (30.1%)	55 (32.9%)	—	—	34 (20.1%)	43 (25.7%)	115 (68.1%)	116 (69.5%)	—	—	—	—	—	—	—	—
Imazio et al., 2014	360	180	180	67.0 (11.1)	68.0 (10.0)	47 (26.1%)	65 (36.1%)	—	—	38 (21.1)	42 (23.3)	121 (67.2%)	122 (67.8%)	49 (27.2%)	54 (30%)	—	—	—	—	Beta blockers: 103 (57.2%) Ace Inhibitors: 88 (48.9%)	Beta blockers: 101 (56.1%) Ace Inhibitors: 100 (55.6%)
Sarzaeem et al., 2014	216	108	108	59.9 (9.3)		31 (28.7%)	29 (26.9%)	—	—	42 (38.9%)	39 (36.1%)	56 (51.9%)	59 (54.6%)	28 (25.9%)	36 (33.3%)	—	—	2 (1.9%)	3 (2.8%)	—	—
Tabbalat et al., 2016	360	179	181	60.8 (45.9)	60.5 (55.6)	38 (21.2%)	38 (21.0%)	28.3 (4.8)	27.5 (5.2)	93 (52.0%)	85 (47%)	120 (67.0%)	112 (61.9%)	38 (21.2%)	40 (22.1%)	46 (25.7%)	39 (21.5%)	—	—	—	—
Zarpelon et al., 2016	140	71	69	61.5 (10.3)	60.3 (8.1)	22 (30.9%)	23 (33.3%)	—	—	42 (59.2%)	30 (43.5%)	—	—	23 (32.4%)	34 (49.3%)	17 (23.9%)	14 (20.3%)	—	—	Beta blockers: 35 (50%) Ace Inhibitors: 33 (46.5%)	Beta blockers: 37 (53.6%) Ace Inhibitors: 30 (43.5%)
Bessissow et al., 2017	100	49	51	68.9 (7.5)	68.3 (7.4)	33 (68.8%)	22 (43.1%)	—	—	8 (16.3%)	9 (17.6%)	21 (42.9%)	34 (66.7%)	—	—	—	—	7 (14.3%)	3 (5.9%)	—	—
Tabbalat et al., 2020	152	81	71	59 (15.2)	59.8 (13.1)	28.4%	18.3%	29.3 (5.4)	29.3 (5.6)	39.50%	49.30%	55.60%	62%	29.60%	19.70%	18.50%	26.80%	—	—	—	—
Masheyekhi et al., 2020	240	120	120	64.1 (9.2)	59.2 (10.1)	12 (42.3%)	22 (43.1%)	27.6 (2.1)	26.3 (1.9)	7 (25%)	12 (22.7%)	20 (69.4%)	35 (67.2%)	3 (11.9%)	8 (15%)	6 (21.6%)	10 (19.3%)	—	—	Beta blockers: 17 (57.7%) Ace Inhibitors: 14 (47.9%)	Beta blockers: 29 (55.8%) Ace Inhibitors: 29 (55.6%)
Shvartz et al., 2022	240	113	127	62 (8.9)	61 (8.2)	83 (73.5%)	97 (76.4%)	29 (4.4)	29 (4.7)	28 (24.7%)	24 (19%)	100 (88.5%)	119 (93.7%)	25 (22.1%)	41 (32.3%)	46 (40.7%)	51 (40%)	2 (1.7%)	2 (1.6%)	Beta blockers: 84 (74.3%) Ace Inhibitors: 69 (61%)	Beta blockers: 95 (74.8%) Ace Inhibitors: 72 (56.7%)
Conen et al., 2023	3209	1608	1601	68·3 (7·3)	68·3 (7·1)	777 (48·3%)	776 (48·5%)	27·0 (5·3)	27·2 (5·4)	301 (18·7%)	294 (18·4%)	836 (52·0%)	832 (52·0%)	—	—	90 (5·6%)	73 (4·6%)	46 (2·9%)	39 (2·4%)	Beta blockers: 232 (14·4%) Ace Inhibitors: 310 (19·3%)	Beta blockers: 224 (14·0%) Ace Inhibitors: 305 (19·1%)
Diakova et al., 2025 (CAFÉ)	140	70	70	62.5 (7.4)	62.3 (8.9)	23 (38.3%)	20 (33.3%)	28.7 (3.6)	29.2 (4.8)	25 (35.7%)	19 (27%)	67 (95%)	66 (95%)	48 (68.6%)	51 (72.9%)	39 (55.7%)	40 (57.1%)	—	—	—	—
Ryffel et al., 2025 (Co‐STAR)	120	60	60	80.8 (5.2)	80.3 (4.7)	11 (15.7%)	12 (17%)	—	—	21 (35%)	15 (25%)	48 (80%)	49 (82%)	—	—	—	—	5 (8%)	0 (0)	Beta blockers: 22 (37%)	Beta blockers: 24 (40%)

### 3.3. Risk of Bias Assessment

Overall, most studies demonstrated a low risk of bias across all domains. Some concerns were noted in a few trials regarding potential deviations from intended interventions and selective reporting. Only one earlier trial (Sarzaeem et al., 2014) showed a high risk of bias due to incomplete outcome reporting. The newly included RCTs were both judged to be low risk across all five domains.

The summary plot shows that 80% of judgments were rated as low risk, fewer than 20% as some concerns, and < 5% as high risk.

The detailed judgments are presented in Supporting Table 1, with visual summaries shown in Supporting Figures 1 and 2.

## 4. Primary Outcome

### 4.1. POAF

**FIGURE 2:**
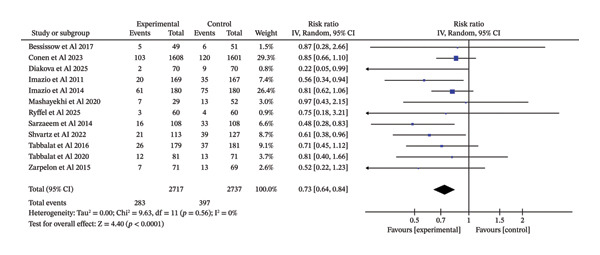
Forest plot showing the pooled effect of colchicine versus control on postoperative atrial fibrillation (POAF). The random‐effects model demonstrated a significant reduction in POAF incidence with colchicine (RR = 0.73, 95% CI = 0.64–0.84, *p* < 0.0001), with zero heterogeneity (I^2^ = 0%). Each square represents an individual study’s risk ratio, and the diamond indicates the overall pooled estimate.

A total of 12 studies (*n* = 5637 participants) were included in the pooled analysis evaluating the effect of colchicine on the incidence of POAF. Colchicine demonstrated a significant reduction in POAF compared to control RR = 0.73 (95% CI = 0.64–0.84; *p* = < 0.0001), with no significant heterogeneity (I^2^ = 0%, *p* = 0.56). Figure 2 Funnel plot inspection revealed a symmetrical distribution of studies, and Egger’s regression test (*z* = −1.69, *p* = 0.09) indicated no evidence of publication bias (Supporting Figure 3). LOO analysis confirmed model robustness, as removal of any single study did not materially alter the overall estimate (pooled RR range = 0.70–0.77; I^2^ < 15%) (Supporting Figure 4).

#### 4.1.1. Subgroup Analysis by Treatment Duration

Upon subgroup analysis by treatment duration, colchicine was found to significantly reduce the risk of POAF for both short‐course regimen (< 2 weeks): RR = 0.72 (95% CI = 0.60–0.85; *p* = 0.0002; I^2^ = 0%) and long‐course regimen (> 2 weeks): RR = 0.77 (95% CI = 0.61–0.96; *p* = 0.02; I^2^ = 0%). No significant subgroup differences were observed between short and long courses (*χ* ^2^ = 0.21; *p* = 0.56) (Supporting Figure 5).

#### 4.1.2. Subgroup Analysis by Type of Surgery

Colchicine demonstrated a significant reduction in POAF incidence across the cardiac surgery subgroup: RR = 0.69 (95% CI = 0.58–0.81; *p* < 0.0001; I^2^ = 0%), but its effects remained nonsignificant across the thoracic surgery subgroup: RR = 0.86 (95% CI = 0.67–1.10; *p* = 0.22; I^2^ = 0%). There was no statistically significant subgroup difference (*χ* ^2^ = 2.09; *p* = 0.15) (Supporting Figure 6).

### 4.2. Secondary Outcomes

#### 4.2.1. GI Events

**FIGURE 3:**
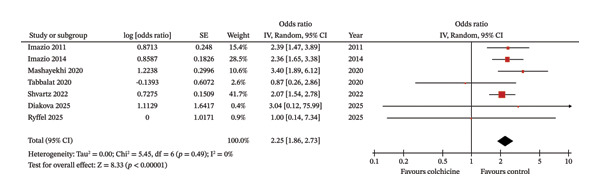
Forest plot showing the pooled effect of colchicine versus control on gastrointestinal (GI) adverse events. The random‐effects model indicated a significantly higher risk of GI events in the colchicine group compared with the control (OR = 2.25, 95% CI = 1.86–2.73, *p* < 0.0001). Heterogeneity was none (I^2^ = 0%). Each square represents the odds ratio for an individual study, with the size proportional to its weight, and the diamond reflects the overall pooled estimate.

Ten studies evaluated GI adverse events. The pooled random‐effects model showed a significant increase in GI events with colchicine OR = 3.11 (95% CI = 2.32–4.16; *p* < 0.0001) with significant heterogeneity (I^2^ = 86%, *p* ≤ 0.00001) (Supporting Figure 7). After sensitivity analysis, removal of high‐variability‐causing studies obtained the refined results with low heterogeneity: OR = 2.25 (95% CI = 1.86–2.73; *p* < 0.0001; I^2^ = 0%). Figure 3 Funnel plot inspection revealed a symmetrical distribution of studies and Egger’s test indicated no small‐study effects (*z* = −1.26; *p* = 0.21) (Supporting Figure 8). LOO analysis confirmed high consistency: 1.12 (95% CI = 0.79–1.44; I^2^ = 89%), suggesting the findings were not driven by any single trial (Supporting Figure 9).

##### 4.2.1.1. Subgroup Analysis by Treatment Duration

Subgroup analysis by treatment duration revealed that short‐course therapy was associated with higher GI event rates: OR = 4.06 (95% CI = 2.88–5.74; *p* < 0.00001), compared with long‐course therapy: OR = 2.54 (95% CI = 1.96–3.29; *p* < 0.00001; *p* for subgroup = 0.03) (Supporting Figure 10).

##### 4.2.1.2. Subgroup Analysis by Type of Surgery

By surgery type, both cardiac: OR = 2.25 (95% CI = 1.86–2.73) and thoracic: OR = 4.30 (95% CI = 2.96–6.33) subgroups showed increased risk of GI events with colchicine (*p* for subgroup = 0.003) (Supporting Figure 11).

#### 4.2.2. Hospital Stay

Six studies assessed length of hospital stay, showing no significant effect of colchicine (MD = −0.60 days; 95% CI = −1.63 to 0.43; *p* = 0.25) with significant heterogeneity (I^2^ = 88%) (Supporting Figure 12). After removing high heterogeneity causing studies, the overall effect remained nonsignificant MD = 0.01 (95% CI = −0.18 to 0.21; *p* = 0.90) while variability became low (I^2^ = 0%) (Supporting Figure 13). Funnel plot and Egger’s test (*t* = −0.58; *p* = 0.59) indicated low likelihood of bias (Supporting Figure 14). The LOO analysis revealed moderate heterogeneity (I^2^ = 87%) yet no single study exerted a disproportionate influence (Supporting Figure 15).

##### 4.2.2.1. Subgroup Analysis by Type of Surgery

Cardiac surgery patients showed a nonsignificant trend toward shorter stays (MD = −1.21; 95% CI = −2.45 to 0.03; *p* = 0.05), whereas thoracic surgery demonstrated no effect (MD = 0.01; 95% CI = −0.19 to 0.21; *p* = 0.94; *p* for subgroup = 0.06) (Supporting Figure 16).

#### 4.2.3. Diarrhea

Six studies reported diarrhea events. Colchicine significantly increased diarrhea incidence (RR = 3.16; 95% CI = 2.25–4.42; *p* < 0.0001), with low heterogeneity (I^2^ = 19%) (Supporting Figure 17). Egger’s test (*z* = −0.57; *p* = 0.57) and funnel plot showed no evidence of publication bias (Supporting Figure 18). The LOO analysis confirmed the stability of results (Supporting Figure 19).

#### 4.2.4. Postoperative Bleeding

Six studies reported postoperative bleeding incidents. The pooled estimate did not show a significant difference between colchicine and control (RR = 0.89; 95% CI = 0.61–1.28; *p* = 0.53), with no heterogeneity (I^2^ = 0%) (Supporting Figure 20). Funnel plot and Egger’s test confirmed absence of bias (*z* = −0.52; *p* = 0.60), and LOO analysis indicated all studies contributed uniformly (*τ* ^2^ = 0) (Supporting Figures 21–22).

#### 4.2.5. Sepsis

Three RCTs evaluated postoperative sepsis. The pooled random‐effects model demonstrated no significant difference between colchicine and control groups (RR = 1.32; 95% CI = 0.48–3.59; *p* = 0.59). Heterogeneity was moderate (I^2^ = 62%; *p* = 0.07) (Supporting Figure 23). After sensitivity analysis, the refined results remained nonsignificant, with no difference in sepsis between the colchicine and placebo groups. RR = 0.77 (95% CI = 0.34–1.74; *p* = 0.53; I^2^ = 0%) (Supporting Figure 24). However, interpretation is limited by the small number of included studies and low event counts.

#### 4.2.6. Hospital Mortality

Five RCTs assessed in‐hospital mortality, finding no significant difference (RR = 0.90; 95% CI = 0.43–1.86; *p* = 0.77; I^2^ = 0%) (Supporting Figure 25). Funnel plot and Egger’s regression test (*z* = 0.69; *p* = 0.49) showed no evidence of publication bias (Supporting Figure 26). Given the rarity of mortality events across included trials, these pooled estimates should be interpreted cautiously.

### 4.3. Certainty Assessment

The certainty of each outcome is presented in Supporting Table 25.

## 5. Discussion

Our meta‐analysis concluded that colchicine therapy significantly reduces the incidence of POAF across cardiac and thoracic surgery groups, without increasing the risk of major complications such as postoperative bleeding, sepsis, or in‐hospital mortality. These findings highlight colchicine’s anti‐inflammatory efficacy and its role in attenuating atrial remodeling and pericardial irritation after surgery. However, these findings were derived from clinically heterogeneous populations including CABG, valve surgery, thoracic surgery, and TAVR cohorts, each characterized by distinct arrhythmic substrates, inflammatory burdens, perioperative pharmacologic strategies, and rhythm‐monitoring intensities. Therefore, although the pooled estimates support a protective effect of colchicine, subgroup‐specific interpretations should be made cautiously.

Our pooled analysis for risk of POAF (RR = 0.73, 95% CI = 0.64–0.84) demonstrated findings similar to prior studies by Imazio et al. (2011, 2014) [23, 24] and Mashayekhi et al. (2020) [31], which reported significant reductions in POAF risk. Notably, our findings also parallel those of Ryffel et al. (2025) [20] and Conen et al. (2023) [22], which confirmed a consistent preventive effect in both cardiac and thoracic surgical groups. These findings are also consistent with prior meta‐analysis by Li et al. [32], which reported a significant reduction in POAF (RR = 0.67; 95% CI = 0.56–0.79) following cardiac surgery. Similar to our findings, Rivera et al. also revealed significant risk reduction for POAF in postsurgical patients [13].

Subgroup analyses demonstrated that both short (< 2 weeks) and prolonged (> 2 weeks) regimens were effective, though short‐term therapy demonstrated slightly greater risk reduction (RR = 0.70 vs. 0.77). This finding is clinically relevant as brief courses minimize GI intolerance while maintaining anti‐inflammatory effects. Rivera et al. (2024) also mirror these findings, showing that POAF risk reduction was not altered by course duration [13]. Zhao et al. (2022) and Ge et al. (2022) reported similar efficacy results, with an overall 35%–40% risk reduction across cardiac and thoracic subgroups [14, 33]. Kommu et al. (2023) found that a perioperative short‐course colchicine regimen yielded a remarkable 30% reduction in POAF incidence after cardiac surgery. It also concluded that a long course did not significantly alter the risk of POAF, rather, it increased GI intolerance [34]. A short‐term colchicine regimen (< 2 weeks) appears optimal, achieving remarkable efficacy with a lower risk of GI intolerance.

Colchicine significantly increased GI adverse events (OR = 2.25; 95% CI = 1.86–2.73) and diarrhea (RR = 3.16; 95% CI = 2.25–4.42). These safety findings are similar to those of the COPPS and COPPS‐2 trials, reporting mild GI intolerance as a prominent dose‐limiting effect [23, 24]. Likewise, Rivera et al. (2024) and Zhao et al. (2022) demonstrated a two‐ to threefold increase in GI adverse events but no serious complications or treatment discontinuations [12, 13]. Kommu et al. (2023) also observed diarrhea as the most frequent adverse effect, yet all events were self‐limited and reversible with temporary dose reduction [34]. The observed heterogeneity in GI adverse events likely reflects clinically important differences among trials, including variation in colchicine dose (0.5–1.0 mg/day), timing of perioperative initiation, treatment duration, use of dose titration strategies, and differences in concomitant perioperative medications such as beta‐blockers or amiodarone. Variability in telemetry protocols and adverse‐event reporting may have also contributed to between‐study differences. Severe complications such as myelosuppression, hepatotoxicity, or neuromyopathy were very rare across trials. This confirms colchicine’s safety at low prophylactic doses (0.5–1 mg/day). Our analysis revealed no significant increase in postoperative bleeding, sepsis, or in‐hospital mortality, further establishing colchicine’s safety and reaffirming its tolerability. Rivera et al. (2024) and Ge et al. (2022) also reported similar results on postoperative infection risk and survival outcomes [13, 33]. The absence of any significant effect on hospital stay further defines that colchicine does not adversely affect perioperative recovery. These results reinforce colchicine’s role as a pleiotropic anti‐inflammatory and anti‐fibrotic agent. It limits pericardial inflammation and atrial oxidative stress, which are key drivers in early POAF. Li et al. (2019) and Kommu et al. (2023) both emphasized that perioperative colchicine reduces inflammatory biomarkers such as CRP and IL‐6, providing a biologic basis for its anti‐arrhythmic efficacy [32, 34]. However, pooled estimates for rare outcomes such as postoperative sepsis and in‐hospital mortality were based on very small event counts across relatively few trials, limiting definitive inference regarding these endpoints.

Collectively, our findings and prior literature converge to support colchicine as a safe, low‐cost, and efficient anti‐inflammatory adjunct for POAF prevention, especially in high‐risk surgical populations. Colchicine stands out as a practical prophylactic option to reduce POAF risk, especially when administered for ≤ 2 weeks in the perioperative period. It demonstrates a favorable balance between efficacy, tolerability, and patient compliance.

### 5.1. Strengths and Limitations

Our meta‐analysis incorporated the most recent RCTs published up to September 2025, making it the largest meta‐analysis exploring the use of colchicine for POAF prevention to date, and it provides space for further research in this area. We utilized random‐effects modeling with the restricted maximum likelihood (REML) estimator and LOO sensitivity analyses to confirm the stability and consistency of results across studies. Our meta‐analysis included diverse surgical settings (both cardiac and thoracic procedures) and varying treatment durations, which allowed us to perform subgroup comparisons, thus broadening the generalizability of findings to real‐world clinical practice.

Nevertheless, certain limitations must be acknowledged. First, clinically meaningful heterogeneity existed across included surgical populations, including CABG, valve surgery, thoracic surgery, and TAVR cohorts, which differ substantially in baseline arrhythmic risk, inflammatory burden, and perioperative management. Although subgroup analyses were performed, thoracic and TAVR subgroup estimates remain underpowered for definitive conclusions. Second, variability in colchicine dosing regimens, timing of perioperative initiation, treatment duration, and concomitant use of beta‐blockers or amiodarone may have contributed to between‐study heterogeneity, particularly for GI outcomes. Third, differences in telemetry protocols, ECG surveillance intensity, and definitions of POAF may have introduced detection bias. Finally, several secondary outcomes, particularly sepsis and mortality, were based on limited event counts and should therefore be interpreted cautiously.

### 5.2. Future Implications and Policy Recommendations

Future research should incorporate short‐course colchicine regimens (< 14 days) at a fixed dosage to further evaluate its efficacy. Guideline committees should consider incorporating colchicine as an adjunctive preventive therapy for high‐risk surgical patients, especially where amiodarone or beta‐blockers are contraindicated. Cost‐effectiveness analyses in low‐resource settings would further support its clinical integration. More studies should be carried out in the thoracic surgery group to further establish colchicine’s role in this particular group.

## 6. Conclusion

This meta‐analysis has shown that colchicine significantly reduces POAF without increasing bleeding, sepsis, or mortality. We also concluded that it is associated with mild, self‐limiting GI adverse effects, but the rarity of any severe adverse events makes it safer and more tolerable. Although both short‐ and long‐course regimens were found to be effective, the short‐course regimen offers an optimal balance between efficacy and patient compliance. These findings support the use of colchicine as a safe and effective adjunct for POAF prevention across surgical populations.

## Author Contributions

Conceptualization: Jarin Rahman, Muhammad Hassan Saeed, Syeda Zaira Haider, and Kishan Chand Lohana; methodology: Muhammad Hassan Saeed, Syeda Zaira Haider, and Muhammad Ahmed Zaheer; literature review: Muhammad Ahmed Zaheer and Safa Mazhar; investigation: Kishan Chand Lohana, Muhammad Ahmed Zaheer, and Safa Mazhar; data curation: Safa Mazhar, Fnu Kashish, and Muhammad Hassan Saeed; formal analysis: Tayyaba Naseem Abbasi, Muhammad Hassan Saeed, and Fnu Kashish; visualization: Tayyaba Naseem Abbasi, Muhammad Hassan Saeed, and Fnu Kashish; writing–original draft: Safa Mazhar, Muhammad Ahmed Zaheer, Syeda Zaira Haider, and Muhammad Hassan Saeed; supporting appendix preparation: Jarin Rahman, Syeda Zaira Haider, and Kishan Chand Lohana; review and editing: Tayyaba Naseem Abbasi, Jarin Rahman, Fnu Kashish, and Kishan Chand Lohana; project administration and supervision: Muhammad Hassan Saeed; and correspondence: Jarin Rahman.

## Funding

No funding or grants were received for this review.

## Ethics Statement

This review did not involve any patients, so no ethical approval was required.

## Conflicts of Interest

The authors declare no conflicts of interest.

## Supporting Information

Additional supporting information can be found online in the Supporting Information section.

## Supporting information


**Supporting Information** Supporting file 1: PRISMA checklist. Supporting file 2: contents are listed below along with relevant page numbers.
