# Supplementary material for: Colchicine Prevents Postoperative Atrial Fibrillation in Cardiac and Thoracic Surgery Patients: Contemporary Evidence From a Meta‐Analysis of Randomized Controlled Trials
Source: Cardiol Res Pract. 2026 Jun 17;2026:1851955. doi: 10.1155/crp/1851955 (PMC13273520; doi:10.1155/crp/1851955)
Supplement: Supplementary file 1 — Supporting Information Supporting file 1: PRISMA checklist. Supporting file 2: contents are listed below along with relevant page numbers. [file CRP-2026-1851955-s001.zip › Supplementary file revised.docx]

**Supplementary Data**

**Index**

**Contents** Page #

**Search Strings** 2

**Supplementary Table 1** 3

**Supplementary Figure 1** 4

**Supplementary Figure 2** 5

**Supplementary Figure 3**  6

**Supplementary Figure 4** 7

**LOO Analysis details POAF** 7-8

**Supplementary Figure 5**  9

**Supplementary Figure 6** 10

**Supplementary Figure 7** 11

**Supplementary Figure 8**  12

**Supplementary Figure 9** 13

**LOO Analysis details GI Events** 13-14

**Supplementary Figure 10** 15

**Supplementary Figure 11** 16

**Supplementary Figure 12** 17

**Supplementary Figure 13**  18

**Supplementary Figure 14** 19

**Supplementary Figure 15** 20

**LOO Analysis details Hospital stay**  20-21

**Supplementary Figure 16** 22

**Supplementary Figure 17** 23

**Supplementary Figure 18**  24

**Supplementary Figure 19** 25

**LOO Analysis details Diarrhea**  25-26

**Supplementary Figure 20** 27

**Supplementary Figure 21** 28

**Supplementary Figure 22** 29

**LOO Analysis details Postoperative Bleeding** 29-30

**Supplementary Figure 23** 31

**Supplementary Figure 24** 32

**Supplementary Figure 25** 33

**Supplementary Figure 26** 34

**Supplementary Table 2** 35

**Search Strategy**

***Search strings***

**Pubmed** = ("Postoperative Atrial Fibrillation" OR ("Atrial Fibrillation" AND ("Postoperative Period" OR "Cardiac Surgical Procedures") OR "postoperative atrial fibrillation" OR "POAF" OR "atrial fibrillation after cardiac surgery" OR "post-cardiac surgery arrhythmia") AND ("Colchicine" OR colchicine OR "anti-inflammatory therapy" OR "NLRP3 inflammasome inhibitor") AND ("Cardiac Surgical Procedures" OR "cardiac surgery" OR "coronary artery bypass graft" OR "CABG" OR "valve surgery" OR "cardiopulmonary bypass") AND ("Randomized Controlled Trial" OR randomized OR randomised OR placebo)

**Embase** = ('postoperative atrial fibrillation'/exp OR 'postoperative atrial fibrillation' OR 'POAF' OR 'atrial fibrillation after cardiac surgery') AND ('colchicine'/exp OR colchicine OR 'antiinflammatory therapy' OR 'nlrp3 inflammasome inhibitor') AND ('cardiac surgery'/exp OR 'coronary artery bypass graft'/exp OR 'valve surgery'/exp OR 'cardiopulmonary bypass'/exp) AND ('randomized controlled trial'/exp OR randomized:ab,ti OR randomised:ab,ti OR 'placebo'/exp)

**Scopus** = ("postoperative atrial fibrillation" OR "POAF" OR "atrial fibrillation after cardiac surgery" OR "post-cardiac surgery arrhythmia") AND (colchicine OR "anti-inflammatory therapy" OR "NLRP3 inflammasome inhibitor") AND ("cardiac surgery" OR "coronary artery bypass graft" OR CABG OR "valve surgery" OR "cardiopulmonary bypass") AND (randomized OR randomised OR "clinical trial" OR placebo)

**Cochrane Library** = ("postoperative atrial fibrillation" OR POAF OR "atrial fibrillation after cardiac surgery") AND (colchicine OR "anti-inflammatory therapy" OR "NLRP3 inflammasome inhibitor") AND ("cardiac surgery" OR "coronary artery bypass graft" OR CABG OR "valve surgery" OR "cardiopulmonary bypass") AND (randomized OR randomised OR "clinical trial" OR placebo)

**Google Scholar** = "postoperative atrial fibrillation" OR POAF OR "atrial fibrillation after cardiac surgery" AND colchicine AND ("cardiac surgery" OR CABG OR "valve surgery" OR "cardiopulmonary bypass") AND (randomized OR randomised OR trial OR placebo)

**ClinicalTrials.gov** = "Postoperative Atrial Fibrillation" OR "Atrial Fibrillation after Cardiac Surgery" OR "POAF" OR "Cardiac Surgery" OR "Coronary Artery Bypass" OR "CABG" OR "Valve Surgery" OR "Cardiopulmonary Bypass" AND "Colchicine"

**Supplementary Table 1. Risk of Bias Assessment using RoB-2 Tool for included Randomized Controlled Trials (RCTs)**

| **Study (Author, Year)** | **D1 (Grade, reason)** | **D2 (Grade, reason)** | **D3 (Grade, reason)** | **D4 (Grade, reason)** | **D5 (Grade, reason)** | **Overall Risk** |
| --- | --- | --- | --- | --- | --- | --- |
| Imazio et al., 2011 | Low risk  (Computer-generated randomization, concealed allocation) | Low risk (double blinded, Intention to treat analysis) | Low risk  (Negligible missing data) | Low risk  (Objective ECG-verified AF detection) | Low risk  (results reported as predefined) | Low risk |
| Imazio et al., 2014 | Low risk  (Adequate random sequence and concealment) | Low risk (double blinded, Intention to treat analysis) | Low risk  (Negligible missing data) | Low risk  (Standardized outcome assessment) | Low risk  (results reported as predefined) | Low risk |
| Sarzaeem et al., 2014 | Low risk  (Adequate random sequence) | Some concerns  (Open label) | Low risk  (Negligible missing data) | Some concerns  (No blinded assessment) | Some concerns  (selective reporting) | High risk |
| Tabbalat et al., 2016 | Low risk  (Adequate random sequence and concealment) | Some concerns  (no blinding) | Low risk  (Negligible missing data) | Some concerns  (No blinded assessment) | Low risk  (results reported as predefined) | Some concerns |
| Zarpelon et al., 2016 | Low risk  (Computer-generated randomization, concealed allocation) | Low risk (double blinded, Intention to treat analysis) | Low risk  (Complete data) | Low risk  (Objective ECG-verified AF detection) | Low risk  (results reported as predefined) | Low risk |
| Bessissow et al., 2017 | Some concerns  (Randomization unclear) | Low risk (double blinded, Intention to treat analysis) | Low risk  (Complete data) | Low risk  (ECG monitoring objective) | Some concerns  (selective reporting) | Some concerns |
| Tabbalat et al., 2020 | Low risk  (Computer-generated randomization, concealed allocation) | Low risk (double blinded, Intention to treat analysis) | Low risk  (Complete data) | Low risk  (ECG monitoring objective) | Low risk  (results reported as predefined) | Low risk |
| Masheyekhi et al., 2020 | Low risk  (Computer-generated randomization, concealed allocation) | Low risk (double blinded, Intention to treat analysis) | Low risk  (Complete data) | Low risk  (Blinded assessors for ECG outcomes) | Low risk  (results reported as predefined) | Low risk |
| Shvartz et al., 2022 | Low risk  (Computer-generated randomization, concealed allocation) | Some concerns  (partial blinding) | Low risk  (Negligible missing data) | Low risk  (ECG monitoring objective) | Low risk  (results reported as predefined) | Some concerns |
| Conen et al., 2023 | Low risk  (Multicenter randomization, adequate concealment) | Low risk (double blinded, Intention to treat analysis) | Low risk  (Full follow-up data available) | Low risk  (Blinding maintained) | Low risk  (results reported as predefined) | Low risk |
| Diakova et al., 2025 (CAFÉ) | Low risk  (Concealed allocation, computer generated) | Low risk (double blinded, Intention to treat analysis) | Low risk  (minimal missing data , withdrawal/loss to follow up, reasons reported properly) | Low risk  (Blinding maintained, trained raters) | Low risk  (results reported as predesigned in trial registry) | Low risk |
| Rhyffel et al., 2025 (Co-STAR) | Low risk  (Stratified block randomization with central concealment) | Low risk (double blinded, Intention to treat analysis) | Low risk  (minimal missing data , withdrawal/loss to follow up, reasons reported properly) | Low risk  (Outcomes measured objectively via continuous ECG) | Low risk  (results reported as predesigned in trial registry) | Low risk |

D1: Bias. Bias arising from the randomization process

D2: Bias due to deviations from intended intervention

D3: Bias due to missing outcome data

D4: Bias in measurement of the outcome

D5: Bias in selection of the reported result

**
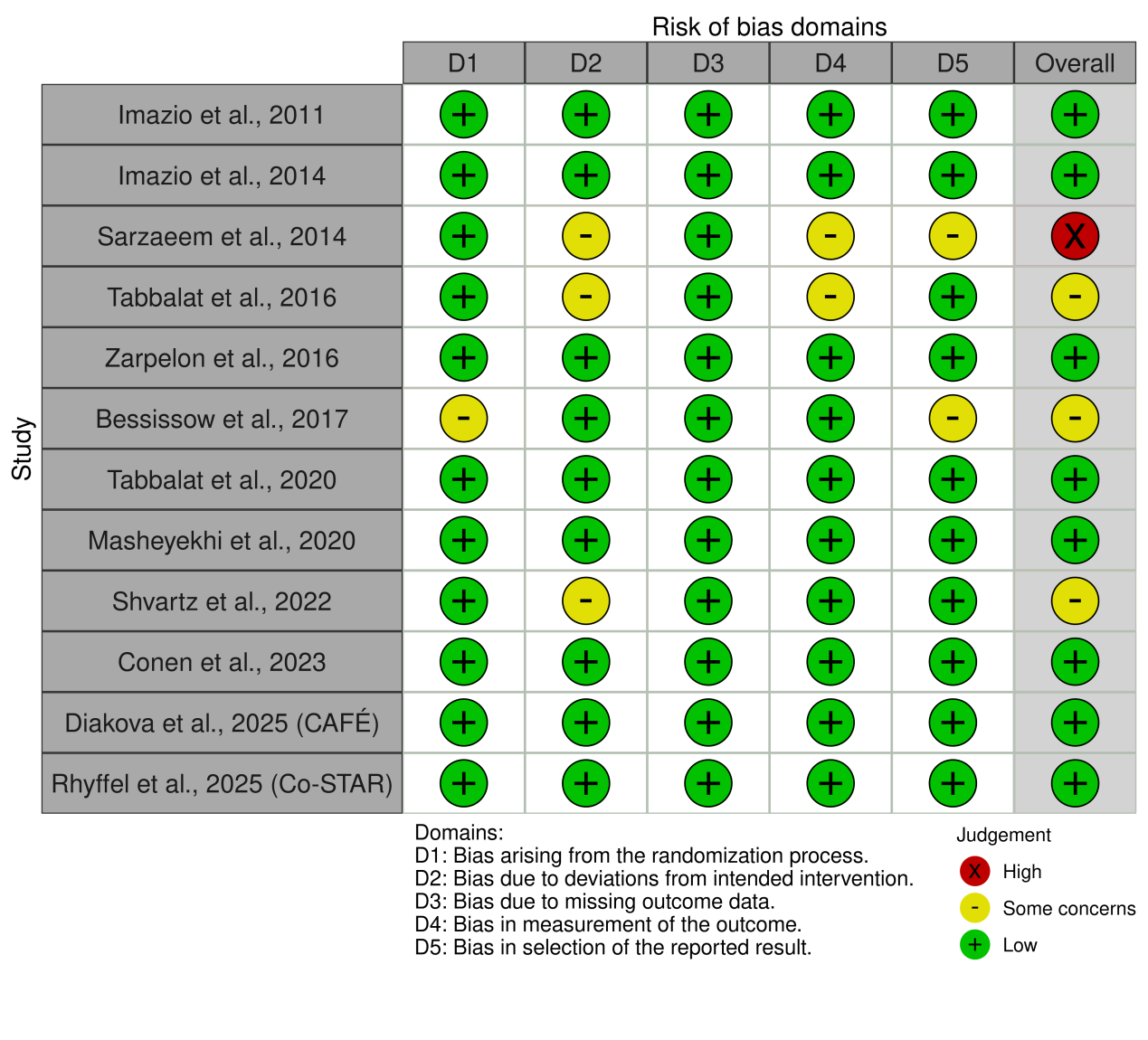
**

**Supplementary Figure 1:** Risk of bias assessment using the Cochrane RoB 2 tool for included randomized controlled trials, illustrated as Traffic light plot.

**
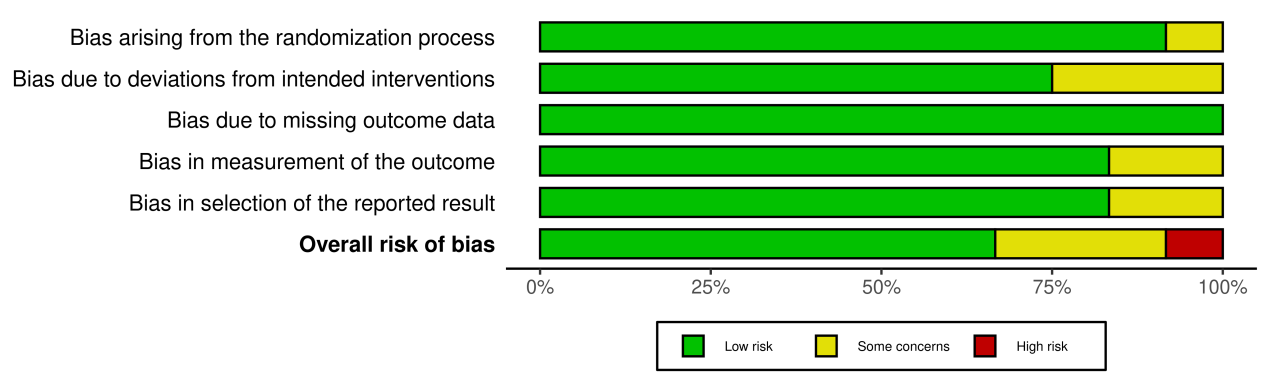
**

**Supplementary Figure 2:**
Summary graph of risk of bias across included randomized controlled trials.

**
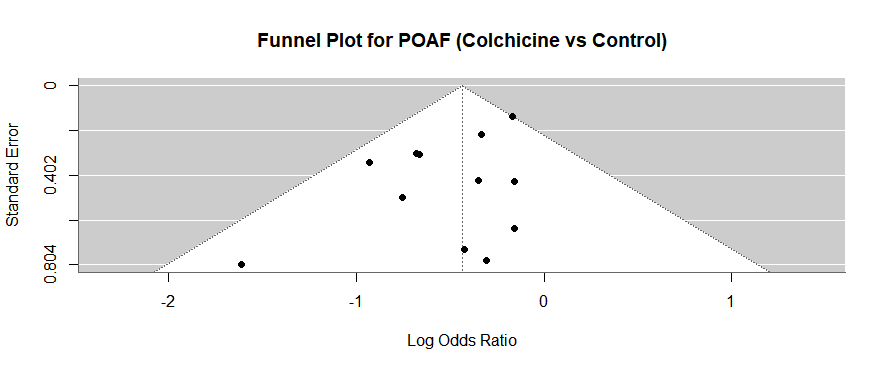
**

**Supplementary Figure 3. Funnel plot assessing publication bias for the effect of colchicine on postoperative atrial fibrillation (POAF).**
The funnel plot demonstrates a symmetrical distribution of studies around the pooled effect size, indicating a low likelihood of publication bias. Egger’s regression test for funnel plot asymmetry was not significant (z = -1.69, p = 0.09), supporting the visual inspection findings.

**
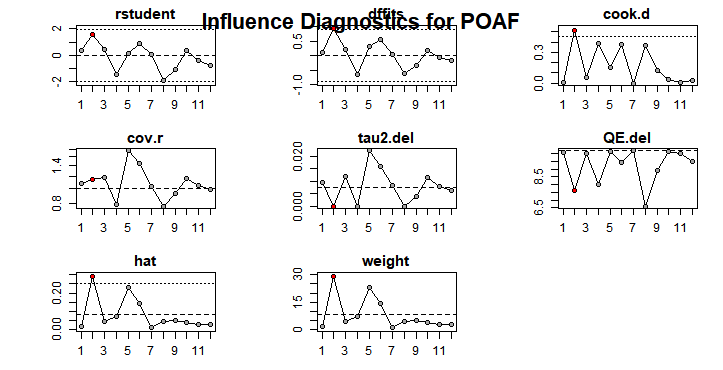
**

**Supplementary Figure 4. Leave-one-out (LOO) influence analysis plot for postoperative atrial fibrillation (POAF).**
Each point represents the pooled effect estimate obtained after sequential omission of one study at a time. The analysis demonstrates that exclusion of any single study does not substantially alter the overall effect size or heterogeneity (I² = 11.7%), confirming the robustness and stability of the meta-analytic model.

LOO ANALYSIS RESULTS FOR POAF : Random-Effects Model (k = 12; tau^2 estimator: REML)

logLik deviance AIC BIC AICc

-2.4859 4.9719 8.9719 9.7677 10.4719

tau^2 (estimated amount of total heterogeneity): 0.0077 (SE = 0.0227)

tau (square root of estimated tau^2 value): 0.0876

I^2 (total heterogeneity / total variability): 11.70%

H^2 (total variability / sampling variability): 1.13

Test for Heterogeneity:

Q(df = 11) = 10.1734, p-val = 0.5149

Model Results:

estimate se zval pval ci.lb ci.ub

-0.3248 0.0749 -4.3370 <.0001 -0.4716 -0.1780 ***

Study estimate se zval pval

Bessissow 2017 -0.3320165 0.07754407 -4.281650 1.855130e-05

Conen 2023 -0.3786669 0.07995585 -4.735950 2.180319e-06

Diakova 2025 -0.3420913 0.08147278 -4.198842 2.682837e-05

Imazio 2011 -0.2783061 0.06645273 -4.188031 2.813854e-05

Imazio 2014 -0.3542274 0.09913213 -3.573286 3.525298e-04

Mashayekhi 2020 -0.3710497 0.09020184 -4.113549 3.896219e-05

Ryffel 2025 -0.3268707 0.07612728 -4.293739 1.756891e-05

Sarzaeem 2014 -0.2794278 0.06553267 -4.263947 2.008471e-05

Shvartz 2022 -0.2989092 0.07197192 -4.153136 3.279493e-05

Tabbalat 2016 -0.3382466 0.08078217 -4.187145 2.824855e-05

Tabbalat 2020 -0.3207325 0.07640226 -4.197946 2.693474e-05

Zarpelon 2015 -0.3125272 0.07436226 -4.202766 2.636736e-05

ci.lb ci.ub Q Qp tau2 I2

-0.4840001 -0.1800329 10.091462 0.4325060 0.009440361 14.940854

-0.5353774 -0.2219563 7.602604 0.6675905 0.000000000 0.000000

-0.5017750 -0.1824076 10.008600 0.4397391 0.012009403 17.781871

-0.4085510 -0.1480611 7.997383 0.6290926 0.000000000 0.000000

-0.5485228 -0.1599320 10.119525 0.4300703 0.022207199 22.705560

-0.5478420 -0.1942573 9.449604 0.4900308 0.015979131 19.823876

-0.4760774 -0.1776639 10.172993 0.4254494 0.008410903 13.620877

-0.4078695 -0.1509862 6.595682 0.7629838 0.000000000 0.000000

-0.4399716 -0.1578468 8.913996 0.5402847 0.003965025 6.597867

-0.4965768 -0.1799165 10.107235 0.4311362 0.011473384 17.172130

-0.4704782 -0.1709869 9.978086 0.4424180 0.008043606 12.861066

-0.4582745 -0.1667798 9.519194 0.4836378 0.006374611 10.472843

H2

1.175653

1.000000

1.216277

1.000000

1.293754

1.247254

1.157687

1.000000

1.070639

1.207323

1.147593

1.116980

**
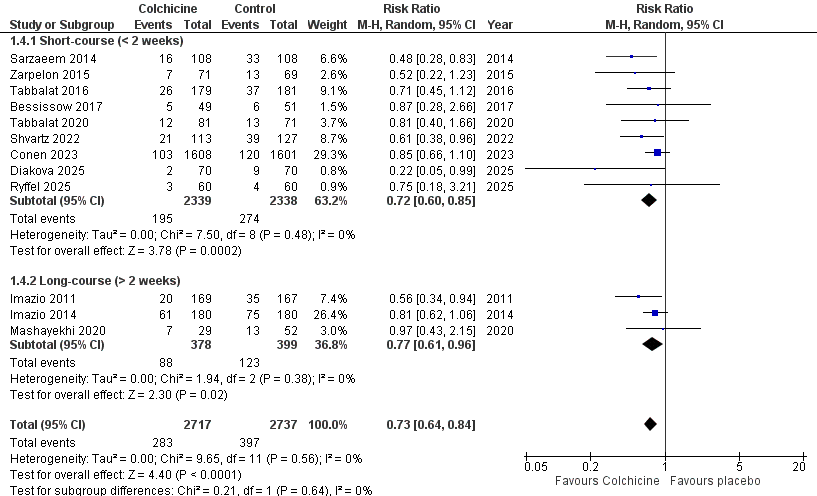
**

**Supplementary Figure 5. Subgroup analysis of colchicine effect on postoperative atrial fibrillation (POAF) by treatment duration.**
Forest plot comparing short-course (< 2 weeks) and long-course (> 2 weeks) colchicine regimens. Both subgroups showed significant reduction in POAF risk, with no significant subgroup difference (χ² = 0.21, p = 0.56), indicating consistent efficacy regardless of treatment duration.

**
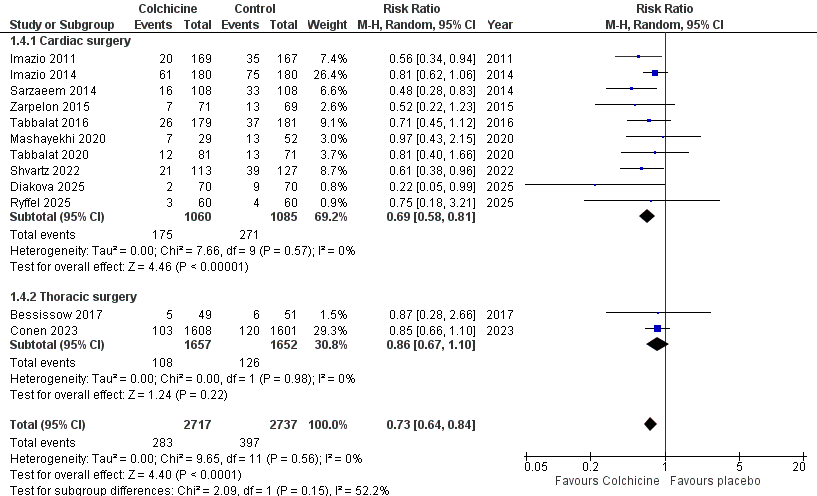
**

**Supplementary Figure 6. Subgroup analysis of colchicine effect on postoperative atrial fibrillation (POAF) by type of surgery.**
Forest plot demonstrating the pooled effect of colchicine across cardiac and thoracic surgery populations. The reduction in POAF was significant in the cardiac subgroup (RR = 0.69, 95% CI 0.58-0.81) and non-significant in the thoracic subgroup (RR = 0.86, 95% CI 0.67-1.10), with no statistically significant subgroup difference (χ² = 2.09, p = 0.15).

**
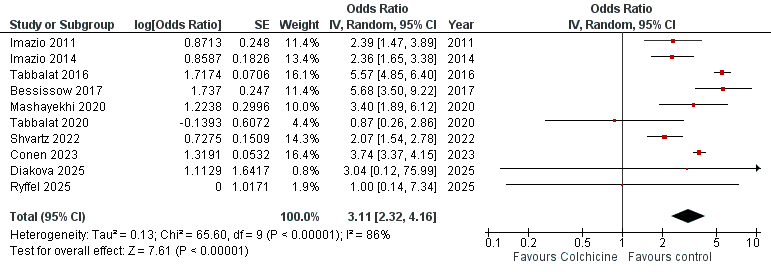
**

**Supplementary Figure 7. Forest plot showing the pooled effect of colchicine versus control on gastrointestinal (GI) adverse events.**
The random-effects model demonstrated a significantly higher risk of GI events among patients receiving colchicine compared with control (OR = 3.11, 95% CI 2.32–4.36, p < 0.0001). Considerable heterogeneity was observed (I² = 86%). Each square denotes an individual study’s odds ratio proportional to its weight, and the diamond represents the overall pooled estimate.

**
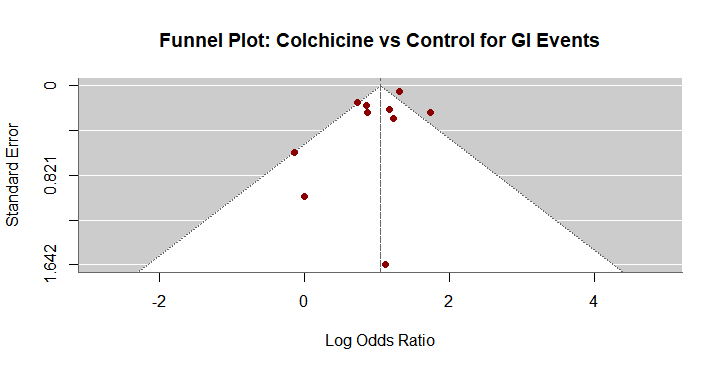
**

**Supplementary Figure 8. Funnel plot assessing publication bias for gastrointestinal (GI) adverse events.**
Visual inspection of the funnel plot revealed a symmetrical distribution of studies, indicating no apparent publication bias. Egger’s regression test confirmed this finding (z = -1.258, p = 0.208), suggesting the absence of small-study effects.

**
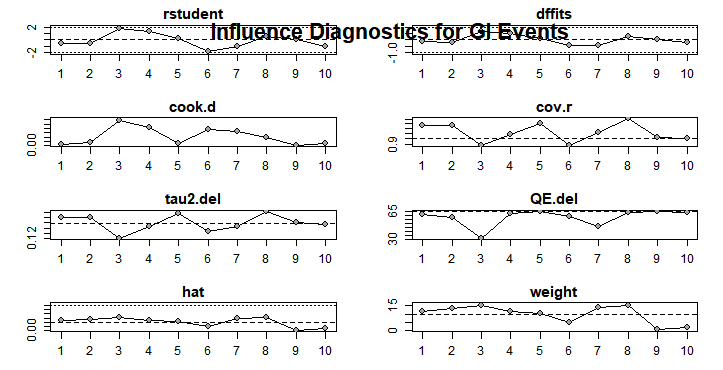
**

**Supplementary Figure 9. Leave-one-out (LOO) influence analysis plot for gastrointestinal (GI) adverse events.**
Sequential omission of individual studies demonstrated that no single trial substantially altered the pooled effect size, confirming the stability of results. Mashayekhi 2020 and Conen 2023 contributed most to heterogeneity, while omission of Tabbalat 2016 produced the largest reduction in I², supporting overall robustness of the analysis.

**LOO ANALYSIS RESULTS FOR GI EVENTS :**

**Random-Effects Model (k = 10; tau^2 estimator: REML)**

**logLik deviance AIC BIC AICc**

**-8.4019 16.8039 20.8039 21.1983 22.8039**

**tau^2 (estimated amount of total heterogeneity): 0.1784 (SE = 0.1206)**

**tau (square root of estimated tau^2 value): 0.4224**

**I^2 (total heterogeneity / total variability): 89.42%**

**H^2 (total variability / sampling variability): 9.45**

**Test for Heterogeneity:**

**Q(df = 9) = 65.6115, p-val < .0001**

**Model Results:**

**estimate se zval pval ci.lb ci.ub**

**1.1165 0.1673 6.6743 <.0001 0.7886 1.4443 *****

**estimate se zval pval ci.lb ci.ub Q**

**-Imazio 2011 1.1410 0.1869 6.1060 0.0000 0.7747 1.5072 61.4942**

**-Imazio 2014 1.1478 0.1877 6.1139 0.0000 0.7799 1.5158 57.4570**

**-Tabbalat 2016 1.0254 0.1571 6.5285 0.0000 0.7175 1.3332 30.8612**

**-Bessissow 2017 1.0392 0.1732 6.0011 0.0000 0.6998 1.3786 63.3209**

**-Mashayekhi 2020 1.0907 0.1914 5.6993 0.0000 0.7156 1.4658 65.3746**

**-Tabbalat 2020 1.1899 0.1580 7.5331 0.0000 0.8803 1.4995 59.4209**

**-Shvartz 2022 1.1842 0.1758 6.7374 0.0000 0.8397 1.5286 46.3172**

**-Conen 2023 1.0655 0.1984 5.3698 0.0000 0.6766 1.4544 63.8093**

**-Diakova 2025 1.1154 0.1693 6.5877 0.0000 0.7836 1.4473 65.5873**

**-Rhyffel 2025 1.1438 0.1677 6.8198 0.0000 0.8151 1.4725 63.7988**

**Qp tau2 I2 H2**

**-Imazio 2011 0.0000 0.2012 91.0441 11.1658**

**-Imazio 2014 0.0000 0.1995 90.5910 10.6281**

**-Tabbalat 2016 0.0001 0.1208 75.5778 4.0946**

**-Bessissow 2017 0.0000 0.1666 89.3763 9.4130**

**-Mashayekhi 2020 0.0000 0.2165 91.7537 12.1266**

**-Tabbalat 2020 0.0000 0.1453 88.4738 8.6759**

**-Shvartz 2022 0.0000 0.1672 88.4930 8.6904**

**-Conen 2023 0.0000 0.2232 83.8923 6.2082**

**-Diakova 2025 0.0000 0.1816 90.6243 10.6659**

**-Rhyffel 2025 0.0000 0.1745 90.2598 10.2667**

**
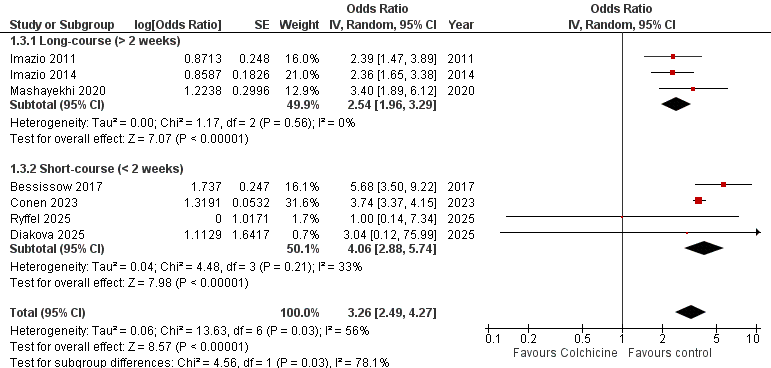
**

**Supplementary Figure 10. Subgroup analysis of gastrointestinal (GI) adverse events by treatment duration.**
Forest plot comparing the incidence of GI events between short-course (< 2 weeks) and long-course (>2 weeks) colchicine regimens. Both subgroups demonstrated a higher risk of GI events with colchicine compared to control, with a greater effect observed in the short-course subgroup (OR = 4.06, 95% CI = 2.88-5.74) than in the long-course subgroup (OR = 2.54, 95% CI = 1.96-3.29), indicating that duration influenced tolerability (p for subgroup = 0.03).

**
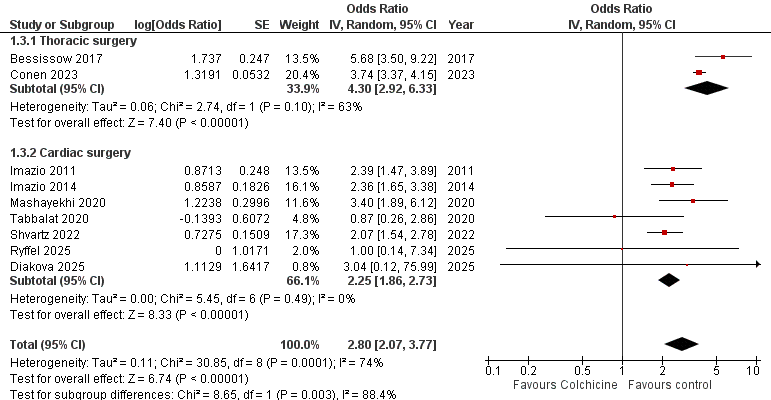
**

**Supplementary Figure 11. Subgroup analysis of gastrointestinal (GI) adverse events by type of surgery.**
Forest plot demonstrating the effect of colchicine on GI adverse events across different surgical types. The increase in GI events was significant in both cardiac (OR = 2.25, 95% CI 1.86-2.73) and thoracic (OR = 4.30, 95% CI 2.96-6.33) surgery subgroups, with a statistically significant subgroup difference (p for subgroup = 0.003), suggesting higher GI intolerance in thoracic surgical patients.

**
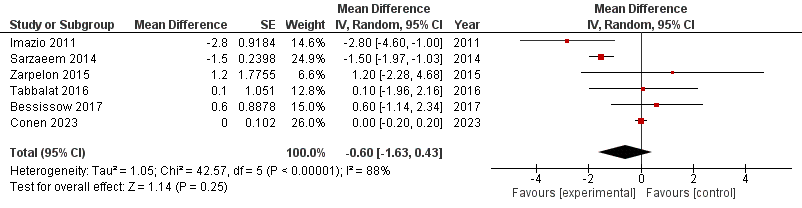
**

**Supplementary Figure 12. Forest plot showing the pooled effect of colchicine versus control on hospital stay duration.**
The random-effects model indicated no significant difference in length of hospital stay between the colchicine and control groups (MD = -0.60 days; 95% CI = -1.63 to 0.43; p = 0.25). Substantial heterogeneity was present (I² = 88%), suggesting variability among included studies. Each square denotes the mean difference for an individual study, sized by study weight, while the diamond represents the overall pooled estimate.

**
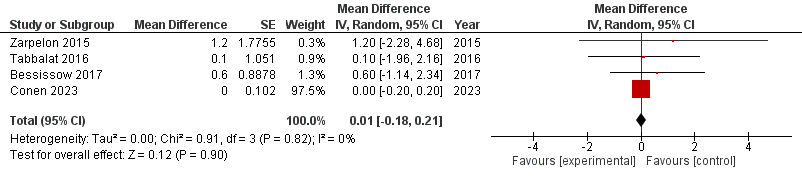
**

**Supplementary Figure 13. Forest plot after sensitivity analysis showing the pooled effect of colchicine versus control on hospital stay duration.**
The random-effects model indicated no significant difference in length of hospital stay between the colchicine and control groups (MD = -0.01 days; 95% CI = -0.18 to 0.21; p = 0.90). Heterogeneity was reduced to 0%. Each square denotes the mean difference for an individual study, sized by study weight, while the diamond represents the overall pooled estimate.

**
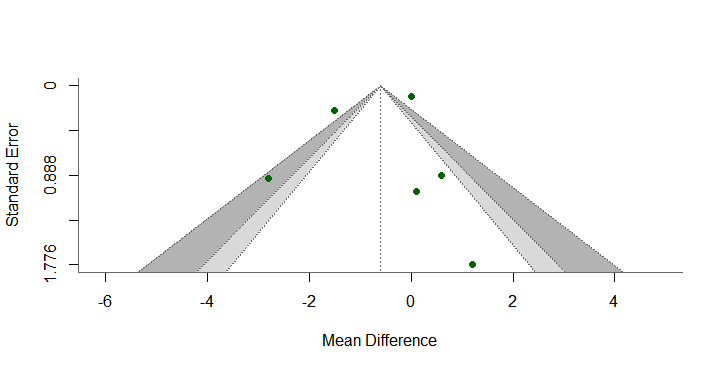
**

**Supplementary Figure 14. Funnel plot assessing publication bias for hospital stay.**
The funnel plot displayed a symmetrical distribution of studies around the pooled mean difference, indicating no evidence of publication bias. Egger’s regression test for funnel plot asymmetry (t = -0.58; p = 0.59) confirmed the absence of small-study effects and supported the overall reliability of the findings.

**
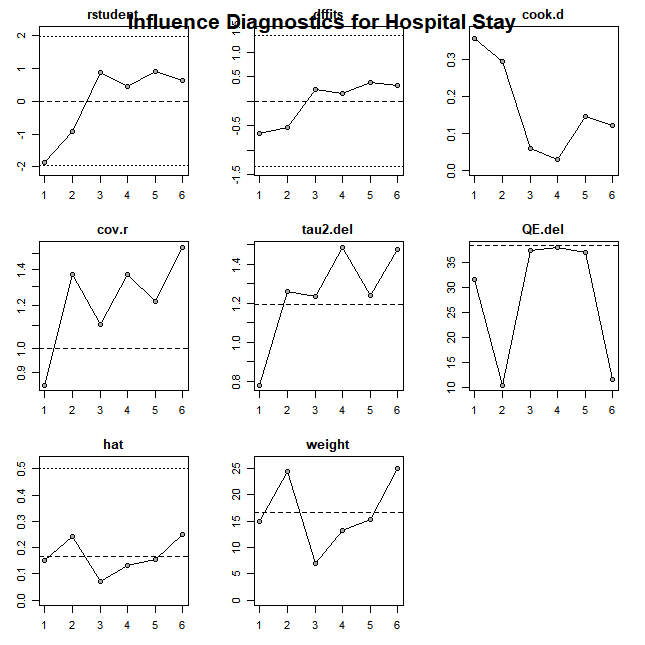
**

**Supplementary Figure 15. Leave-one-out (LOO) influence analysis plot for hospital stay.**
Sequential omission of individual studies demonstrated that no single trial significantly influenced the pooled mean difference or overall significance. Although moderate heterogeneity was observed (I² = 87%), no influential outliers were detected, confirming the robustness and consistency of the findings.

**LOO ANALYSIS DETAILS FOR HOSPITAL STAY :**

**Random-Effects Model (k = 6; tau^2 estimator: REML)**

**logLik deviance AIC BIC AICc**

**-8.9069 17.8138 21.8138 21.0326 27.8138**

**tau^2 (estimated amount of total heterogeneity): 1.1914 (SE = 1.1232)**

**tau (square root of estimated tau^2 value): 1.0915**

**I^2 (total heterogeneity / total variability): 87.09%**

**H^2 (total variability / sampling variability): 7.75**

**Test for Heterogeneity:**

**Q(df = 5) = 38.3070, p-val < .0001**

**Model Results:**

**estimate se zval pval ci.lb ci.ub**

**-0.5705 0.5520 -1.0336 0.3013 -1.6524 0.5114**

**Study estimate se zval pval ci.lb**

**Imazio 2011 -0.2405071 0.5097567 -0.4718077 0.6370640 -1.239612**

**Sarzaeem 2014 -0.2706742 0.6468538 -0.4184473 0.6756201 -1.538484**

**Zarpelon 2015 -0.7042063 0.5806990 -1.2126872 0.2252494 -1.842355**

**Tabbalat 2016 -0.6626853 0.6468488 -1.0244825 0.3056074 -1.930486**

**Bessissow 2017 -0.7819733 0.6099445 -1.2820400 0.1998286 -1.977443**

**Conen 2023 -0.7622794 0.6853119 -1.1123102 0.2660048 -2.105466**

**ci.ub Q Qp tau2 I2 H2**

**0.7585976 31.65640 2.248737e-06 0.780735 83.83960 6.187964**

**0.9971359 10.50930 3.266917e-02 1.258766 68.40860 3.165418**

**0.4339429 37.43019 1.468708e-07 1.235794 89.58826 9.604547**

**0.6051151 38.02067 1.109609e-07 1.487648 90.93990 11.037410**

**0.4134960 36.85362 1.930809e-07 1.242550 89.15300 9.219140**

**0.5809073 11.67163 1.996770e-02 1.475248 70.64579 3.406666**

**
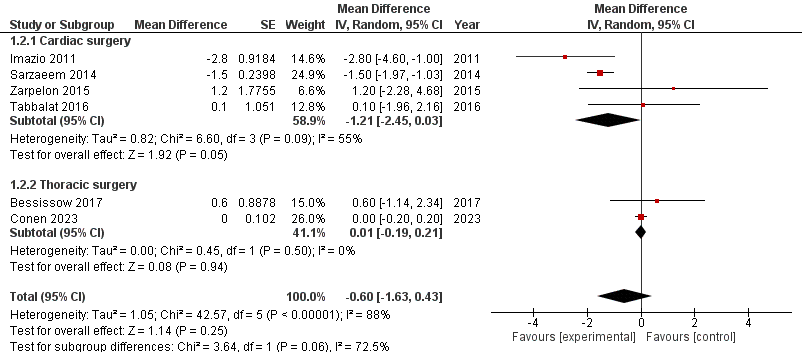
**

**Supplementary Figure 16. Subgroup analysis of colchicine effect on hospital stay by type of surgery.**
Forest plot comparing hospital stay duration between cardiac and thoracic surgery subgroups. A non-significant trend toward shorter hospitalization was observed in cardiac surgery patients (MD = -1.21 days; 95% CI = -2.45 to 0.03; p = 0.05), whereas no difference was seen in thoracic surgery patients (MD = 0.01 days; 95% CI = -0.19 to 0.21; p = 0.94). The difference between subgroups was not statistically significant (p = 0.06).

**
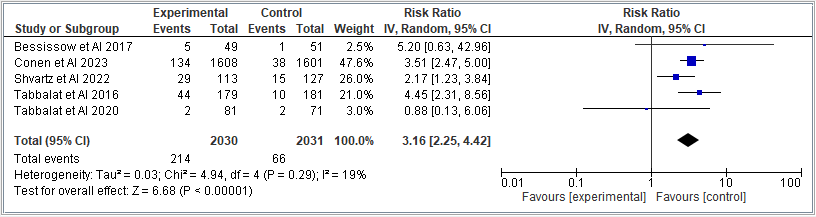
**

**Supplementary Figure 17. Forest plot showing the pooled effect of colchicine versus control on diarrhea incidence.**
Six randomized studies reported diarrhea events. The pooled random-effects model demonstrated a significantly higher risk of diarrhea in the colchicine group (RR = 3.16, 95% CI = 2.25-4.42; p < 0.0001), with low heterogeneity (I² = 19%).

**
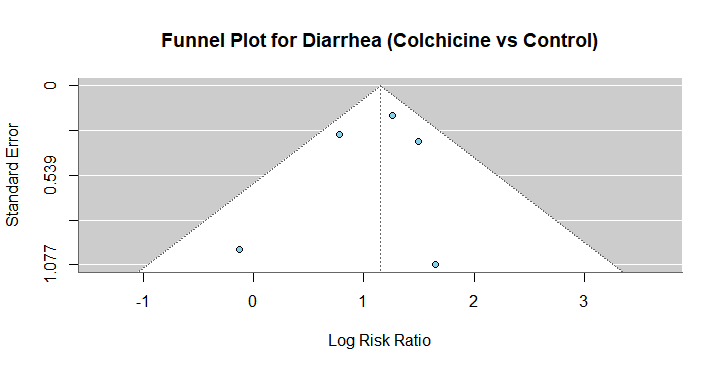
**

**Supplementary Figure 18. Funnel plot assessing publication bias for diarrhea.**
Visual inspection of the funnel plot revealed a symmetrical distribution of studies, indicating no publication bias. Egger’s regression test for funnel plot asymmetry (z = -0.57; p = 0.57) confirmed the absence of small-study effects.

**
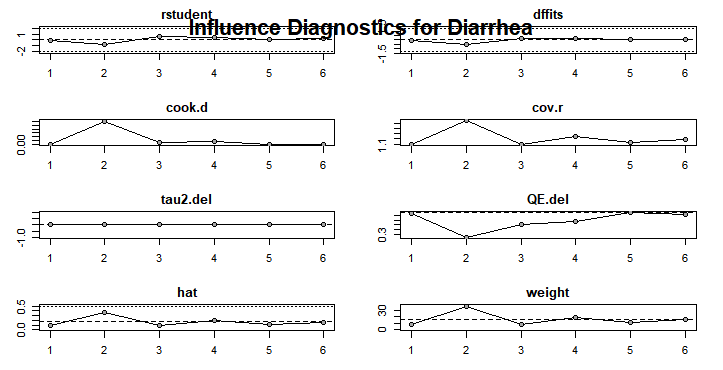
**

**Supplementary Figure 19. Leave-one-out (LOO) influence analysis plot for diarrhea.**
Sequential omission of individual studies showed that no single trial materially altered the pooled estimate or heterogeneity, confirming the stability and robustness of the association between colchicine use and increased diarrhea risk.

**LOO ANALYSIS DETAILS FOR DIARRHEA :**

**Random-Effects Model (k = 6; tau^2 estimator: REML)**

**logLik deviance AIC BIC AICc**

**0.0293 -0.0586 3.9414 3.1602 9.9414**

**tau^2 (estimated amount of total heterogeneity): 0 (SE = 0.0698)**

**tau (square root of estimated tau^2 value): 0**

**I^2 (total heterogeneity / total variability): 0.00%**

**H^2 (total variability / sampling variability): 1.00**

**Test for Heterogeneity:**

**Q(df = 5) = 0.7624, p-val = 0.9794**

**Model Results:**

**estimate se zval pval ci.lb ci.ub**

**0.4677 0.1378 3.3947 0.0007 0.1977 0.7377 *****

**Study estimate se zval pval ci.lb**

**Imazio 2011 0.4735093 0.1440748 3.286551 0.001014223 0.1911278**

**Imazio 2014 0.5431304 0.1728997 3.141303 0.001681979 0.2042532**

**Bessissow 2017 0.4462413 0.1441728 3.095184 0.001966905 0.1636679**

**Conen 2023 0.4386375 0.1535287 2.857038 0.004276147 0.1377266**

**Diakova 2025 0.4674013 0.1459342 3.202822 0.001360881 0.1813755**

**Ryffel 2025 0.4557875 0.1502267 3.033997 0.002413371 0.1613484**

**ci.ub Q Qp tau2 I2 H2**

**0.7558907 0.7433210 0.9458811 0 0 1**

**0.8820076 0.2408599 0.9933051 0 0 1**

**0.7288147 0.5077018 0.9727469 0 0 1**

**0.7395483 0.5786172 0.9654099 0 0 1**

**0.7534271 0.7623808 0.9434191 0 0 1**

**0.7502265 0.7229626 0.9484673 0 0 1**

**rstudent dffits cook.d cov.r tau2.del QE.del hat**

**Imazio 2011 -0.1382 -0.0423 0.0018 1.0936 0.0000 0.7433 0.0856**

**Imazio 2014 -0.7222 -0.5476 0.2999 1.5750 0.0000 0.2409 0.3651**

**Bessissow 2017 0.5047 0.1556 0.0242 1.0951 0.0000 0.5077 0.0868**

**Conen 2023 0.4287 0.2108 0.0445 1.2419 0.0000 0.5786 0.1948**

**Diakova 2025 0.0059 0.0021 0.0000 1.1220 0.0000 0.7624 0.1088**

**Ryffel 2025 0.1986 0.0864 0.0075 1.1890 0.0000 0.7230 0.1590**

**weight dfbs inf**

**Imazio 2011 8.5605 -0.0423**

**Imazio 2014 36.5077 -0.5476**

**Bessissow 2017 8.6847 0.1556**

**Conen 2023 19.4750 0.2108**

**Diakova 2025 10.8758 0.0021**

**Ryffel 2025 15.8963 0.0864**

**
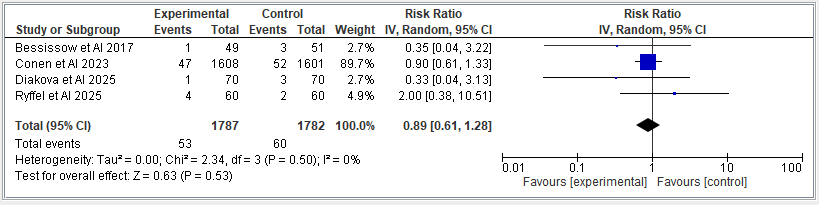
**

**Supplementary Figure 20. Forest plot showing the pooled effect of colchicine versus control on postoperative bleeding.**
Six studies reported postoperative bleeding events. The pooled random-effects model demonstrated no significant difference between colchicine and control groups (RR = 0.89, 95% CI = 0.61-1.28; p = 0.53), with no heterogeneity (I² = 0%).

**
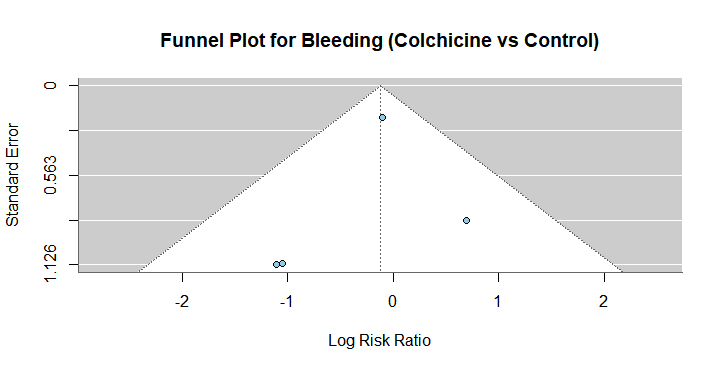
**

**Supplementary Figure 21. Funnel plot assessing publication bias for postoperative bleeding.**
Visual inspection of the funnel plot showed a symmetrical distribution of studies, suggesting no evidence of publication bias. Egger’s regression test confirmed this finding (z = -0.52; p = 0.60).

**
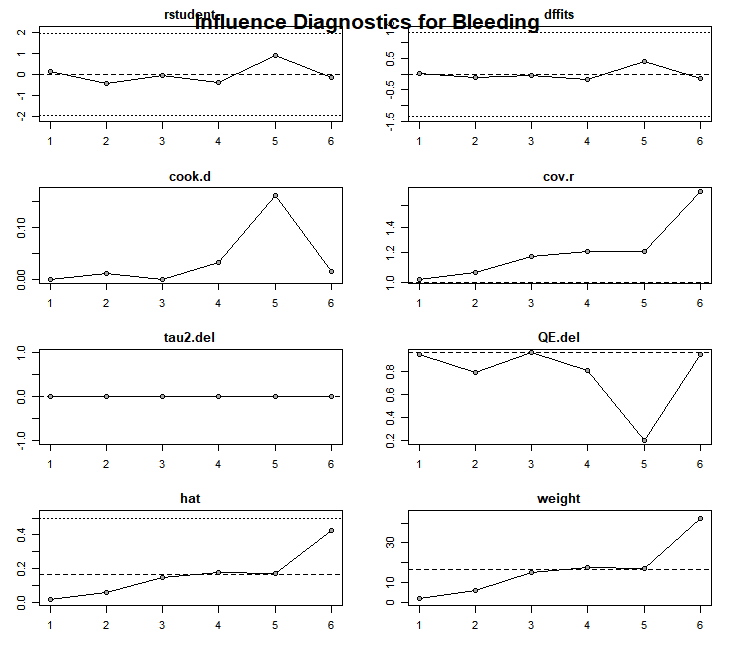
**

**Supplementary Figure 22. Leave-one-out (LOO) influence analysis plot for postoperative bleeding.**
Sequential exclusion of individual studies indicated that all trials contributed uniformly to the pooled estimate, with no influential outliers identified (τ² = 0), confirming the robustness of the analysis.

**LOO ANALYSIS DETAILS FOR POSTOPERATIVE BLEEDING:**

**Random-Effects Model (k = 6; tau^2 estimator: REML)**

**logLik deviance AIC BIC AICc**

**-2.5171 5.0342 9.0342 8.2531 15.0342**

**tau^2 (estimated amount of total heterogeneity): 0 (SE = 0.1340)**

**tau (square root of estimated tau^2 value): 0**

**I^2 (total heterogeneity / total variability): 0.00%**

**H^2 (total variability / sampling variability): 1.00**

**Test for Heterogeneity:**

**Q(df = 5) = 0.9678, p-val = 0.9651**

**Model Results:**

**estimate se zval pval ci.lb ci.ub**

**-0.1896 0.1937 -0.9789 0.3276 -0.5692 0.1900**

**Study estimate se zval pval ci.lb**

**Imazio 2011 -0.1930985 0.1954620 -0.9879078 0.3231978 -0.5761970**

**Sarzaeem 2014 -0.1689367 0.1998254 -0.8454215 0.3978755 -0.5605872**

**Tabbalat 2016 -0.1837578 0.2099159 -0.8753874 0.3813632 -0.5951854**

**Bessissow 2017 -0.1544609 0.2130936 -0.7248504 0.4685438 -0.5721167**

**Shvartz 2022 -0.2673699 0.2129758 -1.2554003 0.2093335 -0.6847949**

**Conen 2023 -0.1647153 0.2556743 -0.6442389 0.5194205 -0.6658277**

**ci.ub Q Qp tau2 I2 H2**

**0.1900001 0.9501508 0.9172582 0 0 1**

**0.2227139 0.7907527 0.9396835 0 0 1**

**0.2276699 0.9625422 0.9154216 0 0 1**

**0.2631948 0.8112424 0.9369345 0 0 1**

**0.1500550 0.1968688 0.9954618 0 0 1**

**0.3363971 0.9455218 0.9179411 0 0 1**

**>**

**rstudent dffits cook.d cov.r tau2.del QE.del hat**

**Imazio 2011 0.1328 0.0180 0.0003 1.0184 0.0000 0.9502 0.0181**

**Sarzaeem 2014 -0.4207 -0.1067 0.0114 1.0644 0.0000 0.7908 0.0605**

**Tabbalat 2016 -0.0723 -0.0302 0.0009 1.1746 0.0000 0.9625 0.1486**

**Bessissow 2017 -0.3956 -0.1815 0.0329 1.2104 0.0000 0.8112 0.1738**

**Shvartz 2022 0.8780 0.4015 0.1612 1.2091 0.0000 0.1969 0.1729**

**Conen 2023 -0.1492 -0.1285 0.0165 1.7425 0.0000 0.9455 0.4261**

**weight dfbs inf**

**Imazio 2011 1.8058 0.0180**

**Sarzaeem 2014 6.0472 -0.1067**

**Tabbalat 2016 14.8627 -0.0302**

**Bessissow 2017 17.3829 -0.1815**

**Shvartz 2022 17.2915 0.4015**

**Conen 2023 42.6099 -0.1285**

**
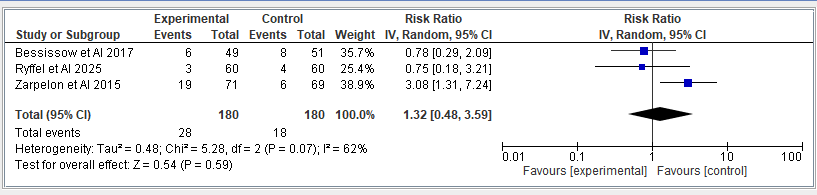
**

**Supplementary Figure 23. Forest plot showing the pooled effect of colchicine versus control on postoperative sepsis**

Three randomized controlled trials evaluated postoperative sepsis. The pooled random-effects model demonstrated no significant difference between colchicine and control groups (RR = 1.32; 95% CI = 0.48-3.59; p = 0.59), with moderate heterogeneity (I² = 62%; p = 0.07).

**
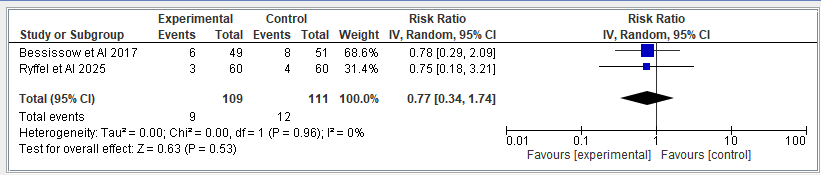
**

**Supplementary Figure 24. Sensitivity analysis for postoperative sepsis.**
After exclusion of the potential outlier study, the refined random-effects model remained non-significant for differences in sepsis incidence between colchicine and placebo groups (RR = 0.77; 95% CI = 0.34-1.74; p = 0.53), with no heterogeneity (I² = 0%), confirming the robustness of the findings.

**
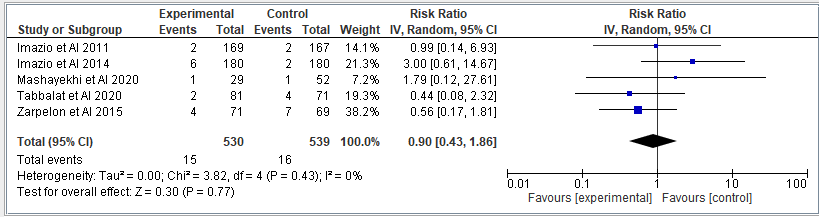
**

**Supplementary Figure 25. Forest plot showing the pooled effect of colchicine versus control on in-hospital mortality.**
Five randomized controlled trials assessed in-hospital mortality. The pooled random-effects model demonstrated no significant difference between colchicine and control groups (RR = 0.90; 95% CI 0.43–1.86; p = 0.77), with no heterogeneity (I² = 0%).

**
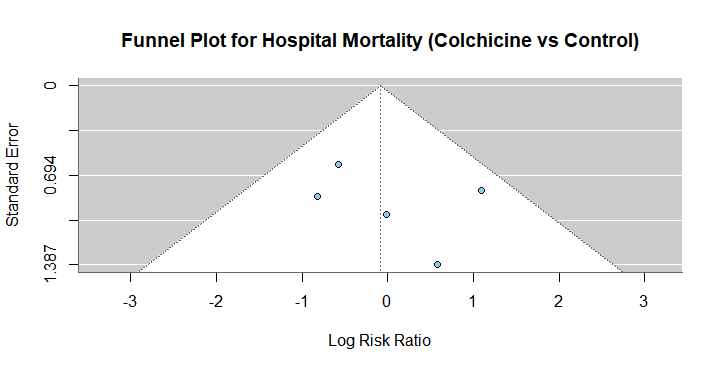
**

**Supplementary Figure 26. Funnel plot assessing publication bias for in-hospital mortality.**
Visual inspection of the funnel plot showed symmetrical study distribution, indicating no evidence of publication bias. Egger’s regression test confirmed this finding (z = 0.69; p = 0.49), supporting the reliability of the pooled mortality estimate.

**Supplementary Table 2. GRADE assessment for certainty of outcomes**

|  | **Total studies and Study design** | **Number of patients** | **Risk of Bias** | **Inconsistency (I^2^)** | **Indirectness** | **Imprecision (95% CI)** | **Pooled Effect**  **(absolute, 95% CI)** | **Certainty** |
| --- | --- | --- | --- | --- | --- | --- | --- | --- |
| **Postoperative Atrial Fibrillation** | 12 RCTs | 5637 | Not serious | Not Serious (0%) | Not Serious | Not serious (0.64-0.84) | RR = 0.73 (0.64-0.84) | High (****) |
| **GI events** | 7 RCTs | 1612 | Not serious | Not serious (0%) | Not Serious | Not Serious (1.86-2.73) | OR = 2.25 (1.86-2.73) | High (******)** |
| **Hospital Stay** | 6 RCTs | 4385 | Not serious | Serious (88%) | Not Serious | Serious  (-1.63 to 0.43) | MD = -0.60  (-1.63 to 0.43) | Low (OO****)** |
| **Diarrhea** | 5 RCTs | 4061 | Not serious | Not Serious  (19%) | Not Serious | Not Serious  (2.25-4.42) | RR = 3.16 (2.25-4.42) | High (******)** |
| **Hospital mortality** | 5 RCTs | 1069 | Not serious | Not Serious  (0%) | Not Serious | Not Serious  (0.43-1.86) | RR = 0.90 (0.43-1.86) | High (******)** |
| **Postoperative Bleeding** | 4 RCTs | 3569 | Not serious | Not Serious  (0%) | Not Serious | Not Serious  **(0.61-1.28)** | RR = **0.89 (0.61-1.28)** | High (******)** |
| **Sepsis** | 3 RCTs | 360 | Not serious | Serious  (62%) | Not Serious | Serious  **(0.48-3.59)** | RR = 1.32  **(0.48-3.59)** | Low (OO****)** |

GI events = Gastrointestinal Events; RCTs = Randomized Controlled Trials; CI = Confidence Interval
